# Risk factors for non-communicable diseases in Afghanistan: insights of the nationwide population-based survey in 2018

**DOI:** 10.1186/s41043-024-00625-0

**Published:** 2024-08-22

**Authors:** Omid Dadras, Muhammad Haroon Stanikzai, Massoma Jafari, Essa Tawfiq

**Affiliations:** 1 Department of Global Public Health and Primary Care, University of Bergen, Bergen, Norway; 2 Department of Public Health, Faculty of Medicine, Kandahar University, District # 10, 3801 Kandahar, Afghanistan; 3 McMaster University, Hamilton, ON Canada; 4 The Kirby Institute, UNSW Sydney, Sydney, Australia

**Keywords:** Non-communicable diseases, NCD, Risk factors, Afghanistan, WHO STEPS

## Abstract

**Background:**

Noncommunicable diseases (NCDs) account for a substantial number of deaths in Afghanistan. Understanding the prevalence and correlates of major NCD risk factors could provide a benchmark for future public health policies and programs to prevent and control NCDs. Therefore, this study aimed to examine the prevalence and correlates of NCD risk factors among adults aged 18–69 years in Afghanistan.

**Methods:**

We used data from the Afghanistan STEPS Survey 2018. The study population were 3650 (1896 males and 1754 females) adults aged 18–69 years sampled from all 34 provinces through a multistage cluster sampling process. Information on behavioural and biological risk factors was collected. We used STATA (version 18.0) for data analysis.

**Results:**

Of the total participants, 42.8% were overweight or obese, 8.6% were current smokers, 26.9% had insufficient physical activities, 82.6% had low consumption of fruits and vegetables, and only 0.5% had ever consumed alcohol. Approximately 15% of participants had a high salt intake, while 25% and 8% had elevated blood pressure and blood glucose levels, respectively. Similarly, around 18% had elevated total cholesterol. The study revealed a lower prevalence of current smoking among females [AOR = 0.17, 95%CI (0.09–0.30)] compared with males, but a higher prevalence in those who had higher education levels [1.95 (1.13–3.36)] compared with those with no formal education. Insufficient physical activity was higher in participants aged 45–69 years [1.96 (1.39–2.76)], females [4.21 (1.98–8.84)], and urban residents [2.38 (1.46–3.88)] but lower in those with higher education levels [0.60 (0.37–0.95)]. Participants in the 25th to 75th wealth percentiles had higher odds of low fruit and vegetable consumption [2.11 (1.39–3.21)], while those in the > 75th wealth percentile had lower odds of high salt intake [0.63 (0.41–0.98)]. Being overweight/obese was more prevalent in participants aged 45–69 years [1.47 (1.03–2.11)], females [1.42 (0.99–2.01)], currently married [3.56 (2.42–5.21)] or ever married [5.28 (2.76–10.11)], and urban residents [1.39 (1.04–1.86)]. Similarly, high waist circumference was more prevalent in participants aged 45–69 years [1.86 (1.21–2.86)], females [5.91 (4.36–8.00)], those being currently married [4.82 (3.12–7.46)], and those being in 25th to 75th wealth percentile [1.76 (1.27–2.43)]. A high prevalence of elevated blood pressure was observed in participants aged 45–69 years [3.60 (2.44–5.31)] and currently married [2.31 (1.24–4.31)] or ever married [6.13 (2.71–13.8)] participants. Elevated blood glucose was more prevalent in older adults ([1.92 (1.09–3.39)] for 45–69 and [3.45 (2.44–5.31)] for 30–44 years), urban residents [2.01 (1.33–3.03)], and ever-married participants [4.89 (1.48–16.2)]. A higher prevalence of elevated cholesterol was observed in females [2.68 (1.49–4.82)] and those currently married [2.57 (1.17–5.63)] or ever married [4.24 (1.31–13.73)].

**Conclusion:**

This study used up-to-date available data from a nationally representative sample and identified the prevalence of NCDs and associated risk factors in Afghanistan. Our findings have the potential to inform and influence health policies by identifying people at high risk of developing NCDs and can assist policymakers, health managers, and clinicians to design and implement targeted health interventions.

## Introduction

According to statistics from 2019, noncommunicable diseases (NCDs) and associated risk factors accounted for 74% of worldwide mortality [1, 2]. An estimated 82–85% of these premature fatalities occur in poor and middle-income countries where people are struggling with economic hardships and weak or unprepared healthcare systems that may be unable to offer long-term services for these diseases [2, 3]. NCDs as chronic diseases provide both a social and health risk due to their high associated costs for the healthcare system, decreased quality of life, and prolonged duration of disease treatment [3]. Thus, recognizing, and mitigating risk factors for NCDs is crucial for improving public health outcomes and for fostering sustainable development and economic resilience.

According to the World Health Organization (WHO), primary risk factors for NCDs or chronic illnesses include tobacco use, lack of physical exercise, hazardous alcohol consumption, poor diets, and polluted air, alongside a mix of genetic, physical, environmental, and behavioural causes [4, 5]. Over the past few decades, NCDs such as diabetes, cardiovascular diseases (CVDs), cancers, and chronic respiratory disease have emerged as the primary cause of death and illness in numerous regions including Afghanistan [1, 6, 7]. Almost 50% of all deaths in Afghanistan are caused by NCDs, and this proportion is projected to rise by 2030 [8]. There is a double burden of communicable and non-communicable illnesses in Afghanistan, made worse by the humanitarian catastrophe that has been triggered by the country’s political instability and armed conflict during the last 40 years [7, 8]. The health situation has been deteriorating, particularly for women, due to widespread vulnerability, which includes extreme poverty, food insecurity, lack of access to safe drinking water and sanitation services, and natural catastrophes such as drought, flooding, and earthquakes [2, 9–11]. Research from 2008 to 2019 shows a sharp increase in Afghanistan’s NCD mortality rates, reaching over 55% for females and 45% for males, with projections indicating that around 60% of female and over 40% of male NCD deaths would be premature by 2030 [2]. In Afghanistan, the availability of NCD treatments is primarily limited to the tertiary level and the private sector, which means that many people (particularly in rural areas) are unable to access these services due to the scarcity of healthcare services and the high cost [8]. A national assessment conducted in 2022 revealed that NCD services were severely disrupted during the COVID-19 pandemic, affecting the availability of diagnostic tools and treatment for chronic illnesses [12].

Globally, studies have consistently demonstrated the significant influence of sociodemographic factors such as age, gender, education, marital status, and residency on the prevalence and management of NCDs [13–15]. Despite these established connections, research in Afghanistan remains sparse, particularly in light of unique societal challenges [16]. The prohibition of women’s education, and severe restrictions on their movement and physical activities [17–19], exacerbate NCD risk factors, compounding the health challenges in the country. Furthermore, since 2021, economic hardships due to reduced international funding have led to decreased family incomes and altered food consumption patterns, which are heavily influenced by the poor economic status in a society [20, 21]. Tobacco use is a major risk factor, contributing to increasing preventable mortality in Afghanistan, affecting more than three million people [11, 22]. Tobacco use also remains prevalent, is culturally accepted, and often is used as a coping mechanism, adding complexity to the public health challenges in the country [22].

This study leverages data from the Afghanistan STEPS survey conducted in 2018, which collected contextualized data on sociodemographic characteristics, beahvioural, and biological factors from household members aged 18–69 years. Although the Afghanistan STEPS survey conducted in 2018 is now six years old, its findings continue to highlight the persistent and potentially worsening situation regarding non-communicable diseases in the region. This highlights a critical research gap and substantiates the need for this study, aiming to provide updated insights and guide effective public health interventions specifically tailored to Afghanistan’s evolving socio-economic conditions.

## Methods

### Data source

This study utilized secondary data from the Afghanistan STEPS survey conducted in 2018 [23]. The survey followed a cross-sectional design using the WHO STEPS approach to collect data on socio-demographic factors, lifestyle, and behaviors, contextualized to Afghanistan. The target population comprised household members aged 18–69 years.

### Sampling

The sampling approach of the STEPS 2018 has been described in detail elsewhere [23]. In brief, the districts served as primary sampling units (PSUs), villages/blocks as secondary sampling units (SSUs), and households within districts as tertiary sampling units (TSUs). The total number of districts in 34 provinces of Afghanistan is 417. From 417 districts, 55 were randomly selected based on resource availability. The total sample size was distributed proportionately to district size. Within each district, enumeration areas (EAs) were selected using the Expanded Program on Immunization (EPI) sampling frame. Households were then randomly selected within each EA, and interviews were conducted with randomly chosen male or female household members. Inclusion criteria were: being permanent residents of the household aged 18–69 and consent to participate in the study. Exclusion criteria were: Temporary residents (residing for less than 12 weeks) of households aged 18–69 or beyond the age limit and refusing to participate in the study. More details on the sampling strategy and sample size calculation are provided in the final report of Afghanistan STEPS 2018 [23].

### Data collection

The data collection process followed WHO's STEPS-wise approach, involving three sequential steps to gather the required information [23]. The first step involved administering a questionnaire (utilizing the STEPS instrument) to gather demographic and behavioral information in a household setting. Data regarding socio-demographic factors, tobacco and alcohol use, dietary behavior, physical activity, history of non-communicable disease (NCD) conditions, and lifestyle advice were collected. In the second step, physical measurements of the respondents were conducted in a household setting. Weight, height, waist circumference, and blood pressure were measured. In the third and final step, blood samples were collected from the respondents in a convenient setting. These samples were used to measure blood glucose, total cholesterol, triglycerides, and High Density Lipoprotein (HDL) cholesterol using dry chemistry methods (blood collection from the fingertip and testing through a strip), with the data recorded accordingly. The use of dry chemistry for blood testing was chosen due to its convenience for participants and its suitability for resource-limited settings such as Afghanistan.

### Measurement and scales

After completing the questionnaire, participants underwent anthropometric measurements conducted by trained healthcare staff in a safe and secure area. If a private area is not available, a separate screened-off area should be set up to ensure privacy for waist and hip circumference measurements. Height and weight were measured using a portable electronic scale and a rigid measuring bar. Waist circumference (WC) was measured at the midpoint between the bottom of the lowest rib and the top of the hip along the mid-axillary line. Blood pressure was measured with a calibrated sphygmomanometer after the participants had been seated for at least 15 min. Three blood pressure readings were taken, with participants resting for three minutes between each reading. For data analysis, the mean of the second and third readings was calculated. Finally, blood samples were collected to assess biochemical markers, including high urinary sodium concentration, elevated blood glucose, and abnormal blood lipid levels. Male household members were interviewed by male interviewers, and female household members were interviewed by female interviewers. More details of the measurement procedure are provided elsewhere [23, 24].

### Study variables

*Sociodemographic characteristics* were age (18–29, 30–44, and 45–69 years), sex (male, female), education (no education, primary/secondary school, high school/higher), marital status (never/currently/ever married), employment (homemakers, unpaid job, self-employed, governmental/NGO employee), income (≤ 25th, 25th to 75th, and ≥ 75th percentiles).

*Behavioral risk factors* were current smoking (yes, no); ever alcohol consumption (yes, no); insufficient physical activity (based on WHO definition, less than 150 min of moderate-intensity physical activity or 75 min of vigorous-intensity physical activity); low fruit and vegetable consumption (taking less than five servings of fruits and/or vegetables on average per day); diet high in salt (based on self-report amount, too much, right amount, too little) [24].

*Biological risk factors* were overweight and obesity (BMI ≥ 25 kg/m2); high waist circumference (based on WHO guidelines a waist circumference of > 79 cm in females and > 89 cm in men) elevated blood pressure (systolic BP ≥ 140 mm Hg or diastolic BP ≥ 90 mm Hg); elevated blood glucose (≥ 7.0 mmol/L or, ≥ 126 mg/dl); elevated cholesterol (≥ 5.0 mmol/L) [24].

### Statistical analysis

**Fig. 1:**
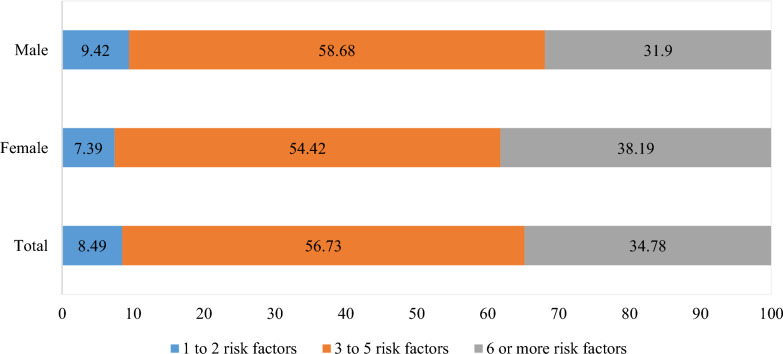
The proportion of combined risk factors, by sex

Descriptive statistics were used to estimate the prevalence of NCD risk factors and the distribution of sociodemographic characteristics across the risk factors. Bivariate analysis (Chi-square test) was employed to examine the association between each sociodemographic characteristic and the risk factors. Multivariable logistic regression was used to examine the odds of outcome across included risk factors. Separate logistic regression models were constructed by including the variables with *p* value < 0.25 in bivariate analysis. The multicollinearity was tested and likelihood ratio tests were used to select the best model for each risk factor. Step-specific sampling weights were applied for behavioral and biological risk factors as recommended by the WHO STEPS survey [24]. The results were presented as frequency and proportion (%) as well as adjusted odds ratio (AOR) for multivariable regression analysis. The sampling design was accounted for in all analyses. The prevalence of combined risk factors was estimated and presented in the frequency histogram in Fig. 1. The analysis was performed in STATA 18. The statistically significant level was determined as *p* < 0.05.

## Results

As Table 1 presented, the largest age group was 18–29 years old (45.4%) followed by 30–44 years old and 45–69 years old. There were slightly more males (51.9%) than females (48.1%). Almost half of the participants lived in rural areas. The majority of participants (59.0%) had no education. The most common marital status was married (76.7%) followed by never married (18.6%). The largest income percentile was the 25th to 75th percentile (39.0%) followed by ≤ 25th percentile (35.0%) and ≥ 75th percentile (26.0%).

**Table 1:** Participants’ characteristics in STEPS 2018

Variables	Frequency (%)
Age (In completed years)	
18–29	1358 (45.4)
30–44	1113 (32.0)
45–69	1179 (22.6)
Sex	
Male	1896 (51.9)
Female	1754 (48.1)
Residential area	
Urban	1891 (51.8)
Rural	1759 (48.2)
Marital status	
Never married	543 (18.6)
Married	2950 (76.7)
Ever married	157 (4.7)
Education	
No formal education	2030 (59.0)
Primary/secondary school	893 (23.5)
High school or higher	727 (17.5)
Income (percentile)	
≤ 25th	1161 (35.0)
25th to 75th	919 (39.0)
≥ 75th	1036 (26.0)

### The prevalence and associated sociodemographic of behavioral NCD risk factors

The prevalence of current smoking, ever drink alcohol, low fruit and vegetable consumption, high salt intake, and insufficient physical activity among adults aged 18–69 years old were 8.6%, 0.5%, 82.6%, 15.1%, and 26.9%, respectively.

Current smoking: In both bivariate (Tables 2, 3) and multivariable analysis (Tables 4), females were significantly less likely to smoke [AOR = 0.17, 95%CI (0.09–0.30)]. Similarly, individuals with higher education were more likely to smoke [AOR = 1.95, 95%CI (1.13–3.36)], as compared with those with no formal education (Table 4).

**Table 2:** The prevalence and association of NCD behavioral risk factors with participants’ background characteristics, Afghanistan STEPS 2018

	Current smoking	Ever drink alcohol	Low fruit & vegetable consumption	High salt intake	Insufficient physical activity
	%	%	%	%	%
Overall	8.6	0.5	82.6	15.1	26.9
Age (year)					
18–29	8.2	0.4	83.7	16.5	22.0
30–44	10.8	0.8	83.5	15.8	27.9
45–69	6.2	0.03	79.3	11.4	35.6
*p* value	*0.20*	*0.26*	*0.21*	*0.10*	< *0.001*
Sex					
Male	14.2	0.7	85.8	14.6	13.9
Female	2.6	0.3	78.2	15.6	41.1
*p* value	< *0.001*	*0.26*	*0.14*	*0.53*	< *0.001*
Residential area					
Rural	7.1	0.4	80.8	15.6	19.0
Urban	9.7	0.6	84.0	14.7	32.8
*p* value	*0.35*	*0.49*	*0.45*	*0.73*	*0.01*
Education					
No education	5.2	0.1	78.8	14.9	32.5
Primary/secondary school	13.0	0.8	88.5	16.9	22.3
High school or higher	14.1	1.3	87.5	14.37	15.0
*p* value	< *0.001*	*0.01*	*0.01*	*0.77*	< *0.001*
Marital status					
Never married	12.3	0.9	85.5	18.9	17.9
Married	7.9	0.4	825	14.5	27.9
Ever married	4.4	0	72.8	9.3	50.5
*p* value	*0.19*	*0.51*	*0.06*	0.15*	< *0.001*
Income (percentile)					
≤ 25th	7.9	0.4	77.2	15.1	26.5
25th to 75th	7.9	0.6	87.3	17.8	31.4
≥ 75th	9.5	0.5	82.8	11.1	27.1
*p* value	*0.84*	*0.84*	*0.03*	*0.02*	*0.51*

Italic value indicates *p*-value of less than 0.05 (*p* < 0.05) is considered statistically significant

**Table 3:** The prevalence and association of NCD biological risk factors with participants’ background characteristics, Afghanistan STEPS 2018

	Overweight and obesity	High waist circumference	Elevated blood pressure	Elevated blood sugar	Elevated cholesterol
	%	%	%	%	%
Overall	42.8	60.7	25.1	8.0	17.5
Age (year)					
18–29	32.3	51.3	15.5	3.9	10.4
30–44	49.9	66.5	22.5	8.2	12.6
45–69	*52.2*	*71.2*	*48.2*	*16.2*	*17.1*
*p* value	< *0.001*	< *0.001*	< *0.001*	< *0.001*	*0.06*
Sex					
Male	38.0	44.1	24.9	6.4	6.9
Female	48.9	78.6	25.2	9.7	18.8
*p* value	*0.002*	< *0.001*	*0.91*	*0.01*	< *0.001*
Residential area					
Rural	39.9	58.1	24.9	5.5	11.3
Urban	45.0	62.6	25.2	10.0	13.6
*p* value	*0.21*	*0.25*	*0.91*	*0.004*	*0.48*
Education					
No education	46.5	69.1	27.0	9.4	15.2
Primary/secondary school	35.4	45.5	24.0	6.9	6.8
High school or higher	41.5	52.8	20.0	4.8	11.7
*p* value	*0.009*	< *0.001*	*0.09*	*0.08*	*0.01*
Marital status					
Never married	18.8	31.6	10.2	3.0	4.6
Married	47.8	66.5	26.9	7.9	13.4
Ever married	64.2	85.0	58.2	30.4	31.2
*p* value	< *0.001*	< *0.001*	< *0.001*	< *0.001*	< *0.001*
Income (percentile)					
≤ 25th	39.9	56.7	25.1	8.3	13.1
25th to 75th	45.5	64.2	24.1	6.4	15.4
≥ 75th	41.5	56.4	25.1	8.7	9.9
*p* value	*0.53*	*0.14*	*0.95*	*0.47*	*0.27*

Italic value indicates *p*-value of less than 0.05 (*p* < 0.05) is considered statistically significant

**Table 4:** The adjusted odds of NCD behavioral risk factors across participants’ background characteristics, Afghanistan STEPS 2018

	Current smoking	Ever drink alcohol	Low fruit & vegetable consumption	High salt intake	Insufficient physical activity
	AOR (95%CI)	AOR (95%CI)	AOR (95%CI)	AOR (95%CI)	AOR (95%CI)
Age (year)					
18–29	Reference	Reference	Reference	Reference	Reference
30–44	1.06 (0.55, 2.63)	1.43 (0.88, 2.31)	1.36 (0.85, 2.16)	1.08 (0.71, 1.65)	1.26 (0.85, 1.89)
45–69	0.82 (0.44, 1.53)	0.72 (0.22, 1.54)	1.06 (0.67, 1.68)	0.80 (0.46, 1.37)	1.96 (1.39, 2.76)
Sex					
Male	Reference	Reference	Reference	–	Reference
Female	0.17 (0.09, 0.30)	0.17 (0.10, 0.30)	0.76 (0.32, 1.78)	–	4.21 (1.98, 8.94)
Residential area					
Rural	–	–	–	–	Reference
Urban	–	–	–	–	2.38 (1.46, 3.88)
Education					
No education	Reference	Reference	Reference	–	Reference
Primary/secondary school	1.66 (0.99, 2.77)	1.74 (1.10, 2.74)	1.58 (0.87, 2.85)	–	0.96 (0.67,1.38)
High school or higher	1.95 (1.13, 3.36)	2.04 (1.19, 3.50)	1.54 (0.78, 3.04)	–	0.60 (0.37, 0.95)
Marital status					
Never married	Reference	–	Reference	Reference	Reference
Married	0.71 (0.31, 1.66)	–	1.31 (0.75, 2.29)	0.72 (0.45, 1.14)	1.25 (0.76, 2.07)
Ever married	1.04 (0.20, 5.33)	–	0.83 (0.32, 2.16)	0.35 (0.11, 1.17)	1.34 (0.60, 3.00)
Income (percentile)					
≤ 25th	–	–	Reference	Reference	–
25th to 75th	–	–	2.11 (1.39, 3.21)	1.15 (0.82, 1.62)	–
≥ 75th	–	–	1.26 (0.63, 2.50)	0.63 (0.41, 0.98)	–

Italic value indicates *p*-value of less than 0.05 (*p* < 0.05) is considered statistically significant

Alcohol consumption: Due to the very low prevalence of ever alcohol consumption, it is difficult to draw reliable conclusions about its association with sociodemographic characteristics. However, higher education and being female were significantly associated with ever alcohol consumption (Table 4).

Low fruit and vegetable consumption: In bivariate analysis, there seems to be a trend towards lower consumption with higher education levels (87.5% for high school or higher and 88.5% for primary or secondary school vs 78.8% for no education) and higher income percentile (82.8% for ≥ 75th percentile and 87.3% for 25th to 75th percentiles vs 77.2% for ≤ 25th percentiles). However, in multivariable analysis, only those in the 25th to 75th percentiles were more likely to have low fruit and vegetable consumption [AOR = 2.11, 95%CI (1.39–3.21)] (Table 4).

High salt intake: Being in ≥ 75th percentiles was associated with a lower rate of high salt intake as compared to being in ≤ 25th percentiles in both bivariate (11% vs 15%%, *p* value = 0.02) and multivariable analysis [AOR = 0.63, 95%CI (0.41–0.98)] (Table 4).

Insufficient physical activity: Participants aged 45–69 years [AOR = 1.96, 95%CI (1.39–2.76)], females [AOR = 4.21, 95%CI (1.98–8.84)], and urban residents [AOR = 2.38, 95%CI (1.46–3.88)] were significantly more likely to be physically inactive while higher education was associated with lower physical inactivity [AOR = 0.60, 95%CI (0.37–0.95)] (Table 4).

### The prevalence and associated sociodemographic of biological NCD risk factors

The prevalence of overweight and obesity, high waist circumference, elevated blood pressure, elevated blood sugar, and elevated cholesterol among adults aged 18–69 years old were 42.8%, 60.7%, 25%, 8%, and 17.5%, respectively.

Overweight and obesity: In both bivariate (Table 3) and multivariable analysis (Table 5), participants aged 45–69 years older age [AOR = 1.47, 95%CI (1.03–2.11)], females [AOR = 1.42, 95%CI (0.99–2.01)], currently married [AOR = 3.56, 95%CI (2.42–5.21)] or ever married [AOR = 5.28, 95%CI (2.76–10.11)], and urban residents [AOR = 1.39, 95%CI (1.04–1.86)] were significantly more likely to be overweight or obese. Despite the higher proportion of overweight among not educated individuals in bivariate analysis, it became insignificant in multivariable analysis (Tables 3 and 5).

**Table 5:** The adjusted odds of NCD biological risk factors across participants’ background characteristics, Afghanistan STEPS 2018

	Overweight and obesity	High waist circumference	Elevated blood pressure	Elevated blood sugar	Elevated cholesterol
	AOR (95%CI)	AOR (95%CI)	AOR (95%CI)	AOR (95%CI)	AOR (95%CI)
Age (year)					
18–29	Reference	Reference	Reference	Reference	Reference
30–44	1.36 (0.99, 1.85)	1.22 (0.82, 1.82)	1.26 (0.81, 1.95)	1.92 (1.09, 3.39)	01.00 (0.57, 1.72)
45–69	*1.47 (1.03, 2.11)*	*1.86 (1.21, 2.86)*	*3.60 (2.44, 5.31)*	*3.45 (1.98, 6.02)*	*1.31 (0.78, 2.20)*
Sex					
Male	Reference	Reference	–	Reference	Reference
Female	1.42 (0.99, 2.01)	5.91 (4.36, 8.00)	–	1.21 (0.74, 1.97)	2.68 (1.49, 4.82)
Residential area					
Rural	Reference	Reference	–	Reference	–
Urban	1.39 (1.04,1.86)	1.13 (0.81, 1.57)	–	2.01 (1.33, 3.03)	–
Education					
No education	Reference	Reference	Reference	Reference	Reference
Primary/secondary school	0.88 (0.62, 1.26)	0.80 (0.56, 1.15)	1.24 (0.89, 1.72)	0.98 (0.43,2.23)	0.68 (0.35, 1.32)
High school or higher	1.11 (0.74, 1.66)	1.00 (0.62, 1.62)	1.04 (0.69, 1.57)	0.65 (0.39, 1.09)	1.23 (0.72, 2.11)
Marital status					
Never married	Reference	Reference	Reference	Reference	Reference
Married	3.56 (2.42, 5.21)	4.82 (3.12, 7.46)	2.31 (1.24, 4.31)	1.55 (0.61, 3.19)	2.57 (1.17, 5.63)
Ever married	5.28 (2.76, 10.11)	2.85 (0.97, 8.40)	6.13 (2.71, 13.87)	4.89 (1.48, 16.22)	4.24 (1.31, 13.73)
Income (percentile)					
≤ 25th	–	Reference	–	–	–
25th to 75th	–	1.76 (1.27, 2.43)	–	–	–
≥ 75th	–	1.35 (0.91, 1.99)	–	–	–

Italic value indicates *p*-value of less than 0.05 (*p* < 0.05) is considered statistically significant

High waist circumference: Similar to overweight and obesity, participants aged 45–69 years [AOR = 1.86, 95%CI (1.21–2.86)], females [AOR = 5.91, 95%CI (4.36–8.00)], those being currently married [AOR = 4.82, 95%CI (3.12–7.46)] were significantly more likely to have high waist circumference. Although income was insignificant in bivariate analysis, being in 25th to 75th percentiles was associated with higher odds of high waist circumference in multivariable analysis [AOR = 1.76, 95%CI (1.27–2.43)]. Similar to overweight and obesity, although a higher proportion of high waist circumference was observed among not educated individuals (69%) in bivariate analysis, it became insignificant in multivariable analysis (Tables 3 and 5).

Elevated blood pressure: In both bivariate and multivariable analyses, age group 45–69 [AOR = 3.60, 95%CI (2.44–5.31)] and currently married [AOR = 2.31, 95%CI (1.24–4.31)] or ever married [AOR = 6.13, 95%CI (2.71–13.8)] were associated with elevated blood pressure (Table 5).

Elevated blood sugar: In bivariate, older adults (16% for 45–69 and 8% for 30–44 vs 22.0% for 18–29), females (10% vs 6% for males), those living in urban areas (10% vs 5,5% in rural areas), and being currently or ever married (respectively 8% and 30% vs 3% for never married) were significantly more likely to have elevated blood sugar. However, in multivariable analysis, only older age ([AOR = 1.92, 95%CI (1.09–3.39)] for 45–69 and [AOR = 3.45, 95%CI (2.44–5.31)] for 30–44 years), living in urban areas [AOR = 2.01, 95%CI (1.33–3.03)], and being ever married [AOR = 4.89, 95%CI (1.48–16.2)] remain significant (Table 5).

Elevated cholesterol: Females [AOR = 2.68, 95%CI (1.49–4.82)] and being currently married [AOR = 2.57, 95%CI (1.17–5.63)] or ever married [AOR = 4.24, 95%CI (1.31–13.73)] were significantly more likely to have elevated cholesterol (Table 5). Older age was marginally associated with elevated cholesterol in bivariate but not in multivariable analysis (Table 3). Although no education (15.2% vs 12% in high school and higher and 7% in primary or secondary school) appeared to be associated with elevated cholesterol, in multivariable analysis became insignificant.

### The combined prevalence of NCD risk factors

As Fig. 1 presents, an estimated 8.5% of adults aged 18–69 years old had 1 or 2 risk factors, whereas the majority (57%) had 3 to 5 or 6 or more (35%) risk factors. A higher proportion of males had 3–5 risk factors compared to females (59% vs 54%) while the proportion of females who had 6 or more risk factors was higher than males (38% vs 32%).

## Discussion

In this study, we reported the prevalence and correlates of risk factors for non-communicable diseases based on a large population-based survey of adults aged 18–69 years in Afghanistan. Overall, the prevalence of most behavioral and biological NCD risk factors was substantial. Moreover, there were significant differences in the prevalence of NCD risk factors across our population according to the different sociodemographic characteristics.

In this study, the prevalence of current smoking (8.6%) was high compared to the findings of most previous studies in Afghanistan (range: 5.1–7.6%) [25–27]. However, several cross-sectional studies and household surveys have yielded higher rates (11–39%) from countries in the region [28–30]. Likewise, a meta-analysis concluded that tobacco smoking will be a major public health problem in most Asian countries in the coming decades [31]. Given the gains of tobacco control in some high-income countries, targeted tobacco control measures, including anti-tobacco public health campaigns, counseling, and regulations, are warranted to reduce tobacco consumption and consequently, smoking-induced health burdens in LMICs, including Afghanistan.

We also observed that the rates for current smoking significantly varied according to the sex and education levels of the participants. As expected, current smoking rates were lower for female participants than males (2.6% vs. 14.2%), and this was consistent with earlier studies in Afghanistan and other countries [22, 25, 27, 29, 30]. However, in contrast to most previous literature, we found that participants with higher educational levels were more likely to smoke than those with no education [29, 32]. An inverse relationship between education and smoking has been documented in several systematic reviews [30, 32]. Therefore, the association between higher educational attainment and tobacco smoking in our settings will be an area for future research.

A growing body of research provides evidence for the protective effect of optimal intake of fruits and vegetables against the risk of NCDs. Yet, a higher percentage (82.6%) of respondents could not meet the current WHO recommendations of eating 5 servings per day, which is higher than those reported in previous studies from Afghanistan [33, 34]. In a meta-analysis of 34 studies from South Asian countries, Afghanistan had the highest adherence levels to these recommendations [33]. Afghanistan is a landlocked country with an agriculture-oriented economy, producing a variety of fruits and vegetables around the year. Due to high transportation costs, local fruit and vegetable growers prefer to supply their products to local markets rather than exporting them to neighboring countries or overseas. This may contribute to the availability of fruits and vegetables in some parts of Afghanistan in the summer and autumn seasons; however, recent flooding and drought in the country highlight serious food security challenges people experience in the country [34]. Natural catastrophes, climate change, and persistent socioeconomic instability spanning over recent years may partially explain the higher rates of low consumption documented in the present study [21]. Another possible explanation for this variation could be due to differences in study design, especially the differences in the timing of data collection, as seasonal effects on the availability, affordability, and consumption of fruits and vegetables in Afghanistan are echoed in an earlier study [34].

Overall, the sociodemographic characteristics, except socioeconomic status, assessed in this study did not explain the variance in fruit and vegetable consumption levels. This finding suggests that cost may be a potential obstacle to optimal intake of fruits and vegetables. Therefore, optimizing fruit and vegetable consumption should be promoted in the Afghan population, and addressing the affordability and availability of these foods is considered a key factor, especially in the socioeconomically disadvantaged populations.

In the present study, high salt intake was 15.1%, and the prevalence significantly differed according to the socioeconomic status of the households. Our finding of excessive salt consumption rates is higher than the rates reported previously from Afghanistan but much lower than those reported in China, Thailand, and Hungary [35, 36]. The estimated average salt intake in the adult Afghan population is reported to be 12.1 gr/day exceeding the WHO recommendations (1–5 gr/day) [37]. Afghans consume a lot of salt due to traditional culinary practices that favor the use of salt for flavor enhancement in various dishes [37]. Additionally, preservation methods for food, especially in regions with limited refrigeration, also contribute to higher salt usage [38]. High salt consumption substantially contributes to NCDs, including hypertension and cardiovascular diseases [36]. WHO urges all countries toward a 30% reduction in average daily salt intake by 2025, which sounds formidable in most settings, precisely in Afghanistan [35]. Although many salt-reducing strategies have been planned for Afghanistan, only a few strategies have been implemented [38, 39]. Moreover, there is a need to systematically investigate the impact of these strategies.

In 2016, the global prevalence of insufficient physical activity stood at 27.9% [40], and the present study also shows that nearly one-third (26.9%) of participants did not meet the current requirements for physical activity. Other studies from Afghanistan reported a similar pattern [41, 42]. Further, our analysis showed that insufficient physical activity is higher among older adults aged 45–69 years, females, and urban residents. However, insufficient physical activity was lower in those with higher educational levels compared to those with no formal education. These factors have been inconsistently reported across studies [41, 43, 44]. Cultural restrictions on women’s physical activities and limited opportunities for exercise may explain the association reported in the current study [41]. For the association of insufficient physical activity with educational attainment, our results align with those reported by studies in other countries [40, 43, 44], which also found an inverse association between high insufficient physical activity levels and high education levels. Given the established health consequence of insufficient physical activity, this study calls for nationwide efforts to promote physical activity in Afghanistan.

Alcohol consumption, with other behavioral risk factors, accounts for a significant proportion of major NCDs. The proportion of individuals consuming alcohol reflects discrepancies in different parts of the world [45, 46]. Moreover, diverse factors, including age, sex, income, employment, marital status, education, religious beliefs, and sociocultural norms and values can influence alcohol consumption levels [46]. In Afghanistan, alcohol drinking is socially unacceptable and religiously viewed as a sin [47]. According to the findings of the present study, a tiny portion (0.5%) of participants reported having consumed alcohol. Likewise, the Afghanistan National Urban Drug Use Study (ANUDUS) reported a national prevalence of alcohol consumption at 0.7% [47]. Therefore, alcohol consumption cannot be considered a potential risk factor for NCDs in Afghanistan, according to the findings of the present study.

Elevated body mass index (BMI) and waist circumference significantly increase the risk of NCDs [48, 49]. In the present study, majority (60.7%) of participants had high waist circumference and nearly half (42.8%) were overweight/obese. These findings imply that like many other developing countries, Afghanistan is also joining the global obesity pandemic, owing to its obesogenic environment. Furthermore, we found that age, sex, and marital status were factors associated with both overweight/obesity and high waist circumference, while wealth percentile was specifically associated with high waist circumference. All these factors have been well documented as risk factors for overweight/obesity and high waist circumference in previous literature [48, 50]. Features of the obesogenic environment, including rapid dietary and lifestyle transition, may contribute to the high prevalence of obesity and high waist circumference in the Afghan population [51]. Additionally, low physical activity, echoed in the current and previous studies, could also explain the high prevalence of these conditions [41]. Specific population-based obesity prevention strategies, focusing on the identified correlates, are urgently needed to tackle the high prevalence of overweight/obesity and high waist circumference in Afghanistan.

Afghanistan, like most other developing countries, is undergoing an epidemiological and nutritional transition [8]. As a result of this rapid change, hypertension has emerged as one of Afghanistan’s pressing health concerns [2, 52]. The data from the STEPS 2018 survey presented herein provided evidence of an alarmingly high prevalence (25%) of high blood pressure among adults in Afghanistan. Earlier studies conducted in Afghan adults had reported hypertension prevalence in the range of 31% to 46.2% [9, 26, 53, 54]. These prevalence rates are alarming, given that high blood pressure is associated with high medical, social, and economic costs. Moreover, we observed that older (45–69 years) and married participants had a higher prevalence of hypertension, which is supported by earlier studies in Afghanistan and elsewhere [9, 26, 55, 56]. Consequently, interventions for reducing the prevalence of high blood pressure should pay more attention to those at greater risk.

We found that elevated blood sugar was prevalent in 8.0% of the sampled population, which is higher than the global prevalence of 6.1% [57]. In our study, we also found that older age, urban residence, and being ever married were strongly associated with higher blood sugar levels. A systematic review from Afghanistan found that advancing age, obesity, and hypertension are the main risk factors for diabetes and the overall prevalence of diabetes was 12.3% [58]. The prevalence of diabetes and its correlates are described in detail elsewhere [59].

High cholesterol level is a major risk factor in the development of cardiovascular diseases. Our study showed that elevated cholesterol occurred in 17.5% of participants, which raises causes for concern and action. However, the prevalence of elevated cholesterol reported in our study is lower than those reported in STEPS surveys in Bangladesh (21.7%) [60], Ethiopia (30.8%) [61], Iran (21.2%) [62], and India (21.6%) [63] but higher than levels reported in Nepal STEPS survey (11%) [64]. The prevalence of elevated cholesterol was significantly higher among females and those being currently or ever married. The association of sex and marital status with elevated cholesterol levels has been consistently reported across epidemiological studies [62–64]. No population-based study reporting the prevalence and correlates of elevated cholesterol in Afghanistan could be found. However, a cross-sectional study among school teachers in Kabul reported a high prevalence of elevated cholesterol (20.0%) [65]. The traditional Afghan diet- often characterized by high usage of animal oils and methods of cooking that result in oily and less healthy food options may account for higher elevated cholesterol levels in the studied population [42].

In this study, we found that 57% and 35% of participants had 3 to 5 and 6 or more risk factors of NCDs, respectively. In a nationally representative survey of 4,073 Bangladeshi adults, the proportions of participants who had 3 to 5 and 6 or more NCD risk factors were 37% and 12%, respectively [66]. Similarly, the prevalence of clustering of NCD risk factors was low in India and Nepal compared to our findings [64, 67]. Our study also demonstrated that the clustering of NCD risk factors was higher in males than females for 3 to 5 risk factors but was lower in males than females for six or more risk factors. To our knowledge, the clustering of NCD risk factors has not been studied in Afghanistan. However, prior studies from other countries have revealed that the clustering of NCD risk factors is higher in males, older adults, urban residents, richest, and illiterates [63, 64, 67]. Overall, the clustering of NCD risk factors is alarmingly high in Afghan adults, highlighting the necessity of targeting the clusters of risk factors for NCDs rather than individual risk factors.

NCDs surveillance system plays an important role in the prevention and control of noncommunicable diseases [68]. However, there are inadequate NCDs surveillance efforts in many LMICs [68, 69]. In Afghanistan, the national NCDs surveillance system, which is crucial for decision-making, has not yet been established [11]. Afghanistan relies heavily on international donors, and recent fluctuation in funding may significantly affect health system [20]. Taken these together, Afghanistan could expect a significant increase in NCDs over the coming years. To tackle the burden of NCDs in Afghanistan, NCDs surveillance efforts should be a key component of the national NCDs prevention and control strategy.

## Strengths and limitations

This study has several strengths. First, this study provided insights, for the first time to our knowledge, about the prevalence and correlates of major risk factors for NCDs using a nationally representative sample. Second, the behavioral and biological correlates examined in the study also had health policy implications and may guide effective public health interventions to reduce the burden of NCDs in Afghanistan. Third, we used a survey questionnaire and study design similar to those applied in the WHO STEPS Survey in LMICs, and this approach has the potential that our findings would be comparable with those reported from other LMICs.

This study also has some limitations. First, the cross-sectional data precludes a temporal relationship between some correlates and NCD risk factors. However, the correlates identified align with previous studies conducted in LMICs. Second, the data in the STEPS survey restricted our evaluation of risk factors for NCDs. Therefore, future studies should consider other risk factors, such as fast-food consumption, sedentary lifestyle, stress, meal patterns, and sleep quality in their analyses, which are equally responsible for the development of NCDs. Third, data on some risk factors were self-reported and are subject to information and recall biases. Some participants may have underreported their behavior on alcohol consumption due to the social and cultural restrictions on alcohol consumption in the country. Another limitation of this study is the potential impact of Afghanistan's evolving sociopolitical situation since 2018 on our findings. Changes in the health infrastructure, economy, and access to health services could significantly alter the study's applicability to the current context.

## Conclusion

This study used up-to-date available data from a nationally representative sample and identified the prevalence of NCDs and associated risk factors in Afghanistan. Our findings have the potential to inform and influence health policies by identifying people at high risk of developing NCDs and undesirable health outcomes and can assist policymakers, health managers, and clinicians to design and implement targeted health interventions and clinical practices with the aims of (i) improving quality of care for people at risk of developing NCDs, (ii) preventing long-term NCD-related sequelae in patients with NCDs, and (iii) reducing the burden of NCDs in Afghanistan.

## Data Availability

WHO STEPwise approach to NCD risk factor surveillance (STEPS) data is a publicly available database at: https://extranet.who.int/ncdsmicrodata upon a reasonable request.

## References

[1] Naghavi M, Ong KL, Aali A, Ababneh HS, Abate YH, Abbafati C, Abbasgholizadeh R, Abbasian M, Abbasi-Kangevari M, Abbastabar H, et al. Global burden of 288 causes of death and life expectancy decomposition in 204 countries and territories and 811 subnational locations, 1990–2021: a systematic analysis for the Global Burden of Disease Study 2021. Lancet. 2024;403(10440):2100–32. 38582094 10.1016/S0140-6736(24)00367-2 PMC11126520

[2] Neyazi N, Mosadeghrad AM, Tajvar M, Safi N. Trend analysis of noncommunicable diseases and their risk factors in Afghanistan. Chronic Dis Transl Med. 2023;9(3):210–21. 37711869 10.1002/cdt3.62 PMC10497825

[3] Siddiqui F, Hewitt C, Jennings H, Coales K, Mazhar L, Boeckmann M, Siddiqi N. Self-management of chronic, non-communicable diseases in South Asian settings: a systematic mixed-studies review. PLOS Glob Public Health. 2024;4(1):e0001668. 38190368 10.1371/journal.pgph.0001668 PMC10773968

[4] World Health Organization. Noncommunicable diseases. https://www.who.int/health-topics/noncommunicable-diseases#tab=tab_1. Accessed Apr 2024.

[5] World Health Organization. Noncommunicable diseases. https://www.who.int/news-room/fact-sheets/detail/noncommunicable-diseases. Accessed Apr 2024.

[6] Rijal A, Adhikari TB, Khan JAM, Berg-Beckhoff G. The economic impact of non-communicable diseases among households in South Asia and their coping strategy: a systematic review. PLoS ONE. 2018;13(11):e0205745. 30462648 10.1371/journal.pone.0205745 PMC6248902

[7] Neyazi N, Mosadeghrad AM, Tajvar M, Safi N. Governance of noncommunicable diseases in Afghanistan. Chronic Dis Transl Med. 2024;10:238–46. 39027200 10.1002/cdt3.118 PMC11252431

[8] Neyazi N, Mosadeghrad AM, AbouZeid A. Non-communicable diseases in Afghanistan: a silent tsunami. Lancet. 2023;401(10393):2035–6. 37330735 10.1016/S0140-6736(23)01071-1

[9] Noormal AS, Winkler V, Bhusari SB, Horstick O, Louis VR, Deckert A, Antia K, Wasko Z, Rai P, Mocruha AF, et al. Prevalence of major non-communicable diseases and their associated risk factors in Afghanistan: a systematic review and meta-analysis. Ther Adv Chronic Dis. 2024;15:20406223241229850. 38362254 10.1177/20406223241229850 PMC10868487

[10] Safi N, Anwari P, Sidhu L, Ibrahimi AG, Rasekh A, Naseri S, Salemi W, Hemachandra N, Chikvaidze P. The need to sustain funding for Afghanistan health system to prevent excess morbidity and mortality. East Mediterr Health J. 2023;29(2):119–25. 36880493 10.26719/emhj.23.017

[11] Neyazi N, Mosadeghrad AM, Afshari M, Isfahani P, Safi N. Strategies to tackle non-communicable diseases in Afghanistan: a scoping review. Front Public Health. 2023;11:982416. 36908476 10.3389/fpubh.2023.982416 PMC9992526

[12] Neyazi N, Lindan C, Perdes S, Ibrahimi AG, Horemans D, Al Afsoor D. The provision and utilization of essential health services in Afghanistan during COVID-19 pandemic. Front Public Health. 2022;10:1097680. 36711388 10.3389/fpubh.2022.1097680 PMC9878336

[13] Phaswana-Mafuya N, Peltzer K, Chirinda W, Musekiwa A, Kose Z. Sociodemographic predictors of multiple non-communicable disease risk factors among older adults in South Africa. Glob Health Action. 2013;6:20680. 24044582 10.3402/gha.v6i0.20680 PMC3776324

[14] Peng W, Chen S, Chen X, Ma Y, Wang T, Sun X, Wang Y, Ding G, Wang Y. Trends in major non-communicable diseases and related risk factors in China 2002–2019: an analysis of nationally representative survey data. Lancet Reg Health West Pac. 2024;43:100809. 38456095 10.1016/j.lanwpc.2023.100809 PMC10920046

[15] da Silva AG, Souza JB, Gomes CS, da Silva TPR, Gomide Nogueira de Sá ACM, Malta DC. Multiple behavioral risk factors for non-communicable diseases among the adolescent population in Brazil: the analysis derived from the Brazilian national survey of school health 2019. BMC Pediatr. 2024;24(1):122. 38360574 10.1186/s12887-024-04601-9 PMC10868108

[16] Stanikzai MH, Wafa MH, Rahimi BA, Sayam H. Conducting health research in the current afghan society: challenges, opportunities, and recommendations. Risk Manag Healthc Policy. 2023;16:2479–83. 38024503 10.2147/RMHP.S441105 PMC10662640

[17] Tawfiq E, Azimi MD, Feroz A, Hadad AS, Soroush MS, Jafari M, Yaftali MS, Saeedzai SA. Predicting maternal healthcare seeking behaviour in Afghanistan: exploring sociodemographic factors and women’s knowledge of severity of illness. BMC Pregnancy Childbirth. 2023;23(1):561. 37533023 10.1186/s12884-023-05750-y PMC10398983

[18] Qaderi S, Miranda AV, Odey GO, Musa SS, Sy Lim LT, Vicente CR, Obnial JC, Ekpenyong A, Negida ASAE, Ahmadi A, et al. Taliban’s war on educating girls and women must end now: a call for global actions. Public Health Chall. 2023;2(2):e80. 10.1002/puh2.80 PMC12039753 40495866

[19] Saboor L, Ahmad L, Ahmad A. The bleak future of Afghan women’s health under the Taliban. Lancet. 2022;399(10328):893–4. 35183298 10.1016/S0140-6736(22)00283-5

[20] Safi N, Anwari P, Safi H. Afghanistan’s health system under the Taliban: key challenges. Lancet. 2022;400(10359):1179–80. 36162414 10.1016/S0140-6736(22)01806-2

[21] Essar MY, Wahdati S, O’Sullivan B, Nemat A, Blanchet K. Cycles of disasters in Afghanistan: the urgent call for global solidarity. PLOS Global Public Health. 2024;4(1):e0002751. 38190415 10.1371/journal.pgph.0002751 PMC10773948

[22] Ahmadi A, Rahimi A, Wardak MF, Ahmadi H, Lucero-Prisno DE 3rd. Tobacco harm reduction in Afghanistan: a recipe for improving smokers’ health. Subst Abuse Treat Prev Policy. 2023;18(1):7. 36683028 10.1186/s13011-023-00517-2 PMC9867861

[23] World Health Organization. STEPS 2018 Afghanistan. https://extranet.who.int/ncdsmicrodata/index.php/catalog/782. Accessed Apr 2024.

[24] World Health Organization. Noncommunicable Disease Surveillance, Monitoring and Reporting. https://www.who.int/teams/noncommunicable-diseases/surveillance/systems-tools/steps/manuals. Accessed Apr 2024.

[25] Alemi Q, Stempel C, Montgomery S. Prevalence and social determinants of tobacco use in Afghanistan. Int Health. 2021;13(1):3–12. 32304214 10.1093/inthealth/ihaa010 PMC7807235

[26] Saeed KMI, Rasooly MH, Brown NJW. Prevalence and predictors of adult hypertension in Kabul, Afghanistan. BMC Public Health. 2014;14(1):386. 24754870 10.1186/1471-2458-14-386 PMC4047782

[27] Saeed KMI. Prevalence of hypertension and associated factors in Jalalabad City, Nangarhar Province, Afghanistan. Cent Asian J Glob Health. 2015;4(1):134. 29138711 10.5195/cajgh.2015.134 PMC5661193

[28] Sreeramareddy CT, Pradhan PMS, Mir IA, Sin S. Smoking and smokeless tobacco use in nine South and Southeast Asian countries: prevalence estimates and social determinants from Demographic and Health Surveys. Popul Health Metrics. 2014;12(1):22. 10.1186/s12963-014-0022-0 PMC4151025 25183954

[29] Basit A, Younus BB, Waris N, Fawwad A. Prevalence of tobacco use in urban and rural areas of Pakistan; a sub-study from second National Diabetes Survey of Pakistan (NDSP) 2016–2017. Pak J Med Sci. 2020;36(4):808–15. 32494279 10.12669/pjms.36.4.1705 PMC7260914

[30] Pahari S, Barman D, Talukdar R. Tobacco usage in India: a meta-analysis of evidence drawn from regional studies between 2010 and 2022. Trop Med Int Health. 2023;28(9):699–709. 37583260 10.1111/tmi.13924

[31] Yang JJ, Yu D, Wen W, Shu X-O, Saito E, Rahman S, Gupta PC, He J, Tsugane S, Xiang Y-B, et al. Tobacco smoking and mortality in Asia: a pooled meta-analysis. JAMA Netw Open. 2019;2(3):e191474–e191474. 30924901 10.1001/jamanetworkopen.2019.1474 PMC6450311

[32] Ahun MN, Lauzon B, Sylvestre M-P, Bergeron-Caron C, Eltonsy S, O’Loughlin J. A systematic review of cigarette smoking trajectories in adolescents. Int J Drug Policy. 2020;83:102838. 32683174 10.1016/j.drugpo.2020.102838

[33] Jayawardena R, Jeyakumar DT, Gamage M, Sooriyaarachchi P, Hills AP. Fruit and vegetable consumption among South Asians: a systematic review and meta-analysis. Diabetes Metab Syndr. 2020;14(6):1791–800. 32947110 10.1016/j.dsx.2020.09.004

[34] Poole N, Amiri H, Amiri SM, Farhank I, Zanello G. Food production and consumption in Bamyan Province, Afghanistan: the challenges of sustainability and seasonality for dietary diversity. Int J Agric Sustain. 2019;17(6):413–30. 10.1080/14735903.2019.1680229

[35] Nie T, Huang S, Yang Y, Hu A, Wang J, Cheng Z, Liu W. A review of the world’s salt reduction policies and strategies - preparing for the upcoming year 2025. Food Funct. 2024;15(6):2836–59. 38414443 10.1039/D3FO03352J

[36] Chen X, Du J, Wu X, Cao W, Sun S. Global burden attributable to high sodium intake from 1990 to 2019. Nutr Metab Cardiovasc Dis. 2021;31(12):3314–21. 34627699 10.1016/j.numecd.2021.08.033

[37] Afroza U, Abrar AK, Nowar A, Akhtar J, Mamun MAA, Sobhan SMM, Cobb L, Ide N, Choudhury SR. Salt intake estimation from urine samples in South Asian population: scoping review. Nutrients. 2023;15(20):4358. 37892433 10.3390/nu15204358 PMC10609745

[38] Buse K, Aftab W, Akhter S, Phuong LB, Chemli H, Dahal M, Feroz A, Hofiani S, Pradhan NA, Anwar I, et al. The state of diet-related NCD policies in Afghanistan, Bangladesh, Nepal, Pakistan, Tunisia and Vietnam: a comparative assessment that introduces a ‘policy cube’ approach. Health Policy Plan. 2020;35(5):503–21. 32091080 10.1093/heapol/czz175 PMC7225567

[39] Ghimire K, Mishra SR, Satheesh G, Neupane D, Sharma A, Panda R, Kallestrup P, McLachlan CS. Salt intake and salt-reduction strategies in South Asia: from evidence to action. J Clin Hypertens (Greenwich). 2021;23(10):1815–29. 34498797 10.1111/jch.14365 PMC8678780

[40] Guthold R, Stevens GA, Riley LM, Bull FC. Worldwide trends in insufficient physical activity from 2001 to 2016: a pooled analysis of 358 population-based surveys with 1·9 million participants. Lancet Glob Health. 2018;6(10):e1077–86. 30193830 10.1016/S2214-109X(18)30357-7

[41] Pengpid S, Noormal AS, Peltzer K. High sedentary behavior and low physical activity among adults in Afghanistan: results from a national cross-sectional survey. Front Public Health. 2023;11:1248639. 37794887 10.3389/fpubh.2023.1248639 PMC10546008

[42] Sahrai MS, Huybrechts I, Biessy C, Rinaldi S, Ferrari P, Wasiq AW, Gunter MJ, Dossus L. Determinants of obesity and metabolic health in the Afghan population: protocol, methodology, and preliminary results. J Epidemiol Glob Health. 2022;12(1):113–23. 34994966 10.1007/s44197-021-00026-0 PMC8907359

[43] Pengpid S, Peltzer K. Prevalence and associated factors of physical inactivity among middle-aged and older adults in India: results of a national cross-sectional community survey. BMJ Open. 2022;12(8):e058156. 36028277 10.1136/bmjopen-2021-058156 PMC9422873

[44] Shiferaw KB, Yalew ES, Zemed A, Yitayal MM, Belay GJ, Alie M, Kibret AK, Takele MD, Abich Y, Gashaw M. Prevalence of physical inactivity and associated factors among older adults in Gondar town, Northwest Ethiopia: a community-based cross-sectional study. BMC Geriatr. 2024;24(1):106. 38287268 10.1186/s12877-024-04701-2 PMC10826078

[45] World Health Organization. Global status report on alcohol and health 2018. https://www.who.int/publications/i/item/9789241565639. Accessed Apr 2024.

[46] Khamis AA, Salleh SZ, Ab Karim MS, Mohd Rom NA, Janasekaran S, Idris A, Abd Rashid RB. Alcohol consumption patterns: a systematic review of demographic and sociocultural influencing factors. Int J Environ Res Public Health. 2022;19(13):8103. 35805782 10.3390/ijerph19138103 PMC9265892

[47] Cottler LB, Ajinkya S, Goldberger BA, Ghani MA, Martin DM, Hu H, Gold MS. Prevalence of drug and alcohol use in urban Afghanistan: epidemiological data from the Afghanistan National Urban Drug Use Study (ANUDUS). Lancet Glob Health. 2014;2(10):e592–600. 25304635 10.1016/S2214-109X(14)70290-6

[48] Ejigu BA, Tiruneh FN. The link between overweight/obesity and noncommunicable diseases in Ethiopia: evidences from nationwide WHO STEPS survey 2015. Int J Hypertens. 2023;2023:2199853. 38023617 10.1155/2023/2199853 PMC10667048

[49] Brauer M, Roth GA, Aravkin AY, Zheng P, Abate KH, Abate YH, Abbafati C, Abbasgholizadeh R, Abbasi MA, Abbasian M, et al. Global burden and strength of evidence for 88 risk factors in 204 countries and 811 subnational locations, 1990–2021: a systematic analysis for the Global Burden of Disease Study 2021. Lancet. 2024;403(10440):2162–203. 38762324 10.1016/S0140-6736(24)00933-4 PMC11120204

[50] Farzadfar F, Yousefi M, Jafari-Khounigh A, Khorrami Z, Haghdoost A, Shadmani FK. Trend and projection of non-communicable diseases risk factors in Iran from 2001 to 2030. Sci Rep. 2024;14(1):8092. 38582931 10.1038/s41598-024-58629-z PMC10998837

[51] Pengpid S, Peltzer K. Underweight and overweight/obesity among adults in Afghanistan: prevalence and correlates from a national survey in 2018. J Health Popul Nutr. 2021;40(1):25. 34090532 10.1186/s41043-021-00251-0 PMC8180065

[52] Stanikzai MH, Wafa MH, Tawfiq E, Jafari M, Le CN, Wasiq AW, Rahimi BA, Baray AH, Ageru TA, Suwanbamrung C. Predictors of non-adherence to antihypertensive medications: a cross-sectional study from a regional hospital in Afghanistan. PLoS ONE. 2023;18(12):e0295246. 38150430 10.1371/journal.pone.0295246 PMC10752561

[53] Saeed KM. Burden of hypertension in the capital of Afghanistan: a cross-sectional study in Kabul City, 2015. Int J Hypertens. 2017;2017:3483872. 28127468 10.1155/2017/3483872 PMC5239979

[54] Baray AH, Stanikzai MH, Wafa MH, Akbari K. High prevalence of uncontrolled hypertension among Afghan hypertensive patients: a multicenter cross-sectional study. Integr Blood Press Control. 2023;16:23–35. 37426064 10.2147/IBPC.S417205 PMC10329427

[55] Mills KT, Bundy JD, Kelly TN, Reed JE, Kearney PM, Reynolds K, Chen J, He J. Global disparities of hypertension prevalence and control. Circulation. 2016;134(6):441–50. 27502908 10.1161/CIRCULATIONAHA.115.018912 PMC4979614

[56] Solomon M, Shiferaw BZ, Tarekegn TT, GebreEyesus FA, Mengist ST, Mammo M, Mewahegn AA, Mengiste BT, Terefe TF. Prevalence and associated factors of hypertension among adults in Gurage Zone, Southwest Ethiopia, 2022. SAGE Open Nursing. 2023;9:23779608231153470. 36761364 10.1177/23779608231153473 PMC9903024

[57] Ong KL, Stafford LK, McLaughlin SA, Boyko EJ, Vollset SE, Smith AE, Dalton BE, Duprey J, Cruz JA, Hagins H, et al. Global, regional, and national burden of diabetes from 1990 to 2021, with projections of prevalence to 2050: a systematic analysis for the Global Burden of Disease Study 2021. Lancet. 2023;402(10397):203–34. 37356446 10.1016/S0140-6736(23)01301-6 PMC10364581

[58] Akhtar S, Nasir JA, Javed A, Saleem M, Sajjad S, Khan M, Wadood A, Saeed K. The prevalence of diabetes in Afghanistan: a systematic review and meta-analysis. BMC Public Health. 2021;21(1):941. 34001088 10.1186/s12889-021-10993-5 PMC8130421

[59] Dadras O, Nyaboke Ongosi A, Wang CW. Prevalence and correlates of diabetes and impaired fasting glucose among adults in Afghanistan: insights from a national survey. SAGE Open Med. 2024;12:20503121241238148. 38516644 10.1177/20503121241238147 PMC10956149

[60] Peltzer K. Prevalence and correlates of dyslipidemia, awareness, and management among adults in Bangladesh in 2018. Popul Med. 2023;5(June):1–9. 10.18332/popmed/167806

[61] Gebreegziabiher G, Belachew T, Mehari K, Tamiru D. Prevalence of dyslipidemia and associated risk factors among adult residents of Mekelle City, Northern Ethiopia. PLoS ONE. 2021;16(2):e0243103. 33561153 10.1371/journal.pone.0243103 PMC7872241

[62] Khanali J, Ghasemi E, Rashidi MM, Ahmadi N, Ghamari SH, Azangou-Khyavy M, Malekpour MR, Abbasi-Kangevari M, Hashemi SM, Naderian M, et al. Prevalence of plasma lipid abnormalities and associated risk factors among Iranian adults based on the findings from STEPs survey 2021. Sci Rep. 2023;13(1):15499. 37726324 10.1038/s41598-023-42341-5 PMC10509214

[63] Thakur JS, Jeet G, Pal A, Singh S, Singh A, Deepti SS, Lal M, Gupta S, Prasad R, Jain S, et al. Profile of risk factors for non-communicable diseases in Punjab, Northern India: results of a state-wide STEPS survey. PLoS ONE. 2016;11(7):e0157705. 27389020 10.1371/journal.pone.0157705 PMC4936739

[64] Bista B, Dhimal M, Bhattarai S, Neupane T, Xu YY, Pandey AR, Townsend N, Gyanwali P, Jha AK. Prevalence of non-communicable diseases risk factors and their determinants: results from STEPS survey 2019. Nepal PLoS One. 2021;16(7):e0253605. 34329300 10.1371/journal.pone.0253605 PMC8323895

[65] Alemi S, Nakamura K, Arab AS, Mashal MO, Tashiro Y, Seino K, Hemat S. Prevalence, determinants, and association of overweight/obesity with non-communicable disease-related biomedical indicators: a cross-sectional study in schoolteachers in Kabul, Afghanistan. PLOS Global Public Health. 2023;3(3):e0001676. 36963052 10.1371/journal.pgph.0001676 PMC10021827

[66] Zaman MM, Bhuiyan MR, Karim MN, Zaman M, Rahman MM, Akanda AW, Fernando T. Clustering of non-communicable diseases risk factors in Bangladeshi adults: an analysis of STEPS survey 2013. BMC Public Health. 2015;15(1):659. 26169788 10.1186/s12889-015-1938-4 PMC4501055

[67] Sarveswaran G, Kulothungan V, Mathur P. Clustering of noncommunicable disease risk factors among adults (18–69 years) in rural population, South-India. Diabetes Metab Syndr. 2020;14(5):1005–14. 32623362 10.1016/j.dsx.2020.05.042

[68] Li P, Ma L, Liu J, Zhang L. Surveillance of noncommunicable diseases: opportunities in the era of Big Data. Health Data Sci. 2022;2022:9893703. 38487489 10.34133/2022/9893703 PMC10878401

[69] Ngaruiya C, Bernstein R, Leff R, Wallace L, Agrawal P, Selvam A, Hersey D, Hayward A. Systematic review on chronic non-communicable disease in disaster settings. BMC Public Health. 2022;22(1):1234. 35729507 10.1186/s12889-022-13399-z PMC9210736

